# Human Mesenchymal Stem Cells-Derived Exosome Mimetic Vesicles Regulation of the MAPK Pathway and ROS Levels Inhibits Glucocorticoid-Induced Apoptosis in Osteoblasts

**DOI:** 10.1155/2023/5537610

**Published:** 2023-09-20

**Authors:** Hongxu Lu, Zhaoxia Zhang, Zhaoying Wang, Jinkui Wang, Tao Mi, Liming Jin, Xin Wu, Junyi Luo, Yimeng Liu, Junhong Liu, Wenquan Cai, Peng Guo, Dawei He

**Affiliations:** ^1^Department of Urology, Children's Hospital of Chongqing Medical University, Chongqing 400014, China; ^2^Chongqing Key Laboratory of Children Urogenital Development and Tissue Engineering, Chongqing 400014, China; ^3^China International Science and Technology Cooperation Base of Child Development and Critical, National Clinical Research Center for Child Health and Disorders, Chongqing, China; ^4^Ministry of Education Key Laboratory of Child Development and Disorders, Chongqing Key Laboratory of Pediatrics, Chongqing 400014, China; ^5^Department of Orthopaedics, Children's Hospital of Chongqing Medical University, Chongqing, China; ^6^Institute of Basic Medicine and Cancer (IBMC), Chinese Academy of Sciences, Hangzhou, Zhejiang 310022, China

## Abstract

**Background:**

Long-term extensive use of glucocorticoids will lead to hormonal necrosis of the femoral head, and osteoblasts play an important role in the prevention of osteonecrosis. However, there is no complete cure for necrosis of the femoral head. Mesenchymal stem cell- (MSCs-) derived exosomes are widely used for the repair of various tissue lesions. Therefore, the aim of this study was to investigate the mechanism of dexamethasone- (DEX-) induced osteoblast apoptosis and the therapeutic effect of human umbilical cord MSC- (hucMSC-) derived exosome mimetic vesicles (EMVs) on osteoblast-induced apoptosis by DEX.

**Methods:**

The viability and apoptosis of primary MC3T3-E1 cells were determined by the Cell Counting Kit-8 (CCK-8), FITC-Annexin V/PI staining and immunoblot. The intracellular levels of reactive oxygen species (ROS) after DEX treatment were measured by 2′, 7′ -dichlorodihydrofluorescein diacetate (DCFH-DA) staining. In this study, hucMSC-EMVs and *N*-acetyl-l-cysteine (NAC) were used as therapeutic measures. The expression of B-cell lymphoma 2-associated X, Bcl 2, HO-1, and nuclear factor erythroid-derived 2-like 2 and MAPK- signaling pathway in osteogenic cell MC3T3-E1 cells treated with Dex was analyzed by the immunoblotting.

**Results:**

DEX significantly induced osteoblasts MC3T3-E1 apoptosis and ROS accumulation. MAPK-signaling pathway was activated in MC3T3-E1 after DEX treatment. hucMSC-EMVs intervention significantly downregulated DEX-induced MAPK-signaling pathway activation and ROS accumulation. In addition, hucMSC-EMVs can reduce the apoptosis levels in osteoblast MC3T3-E1 cells induced by DEX.

**Conclusions:**

Our study confirmed that hucMSC-EMVs regulates MAPK-signaling pathway and ROS levels to inhibit DEX-induced osteoblast apoptosis.

## 1. Introduction

Nontraumatic osteonecrosis of the femeral head (NONFH) is the ischemic necrosis or aseptic necrosis of the femoral head due to various nontraumatic factors [1, 2]. Among them, steroid hormones overuse is the most common nontraumatic cause of osteonecrosis of the femoral head (ONFH) [3]. Epidemiological studies in East Asia showed that nontraumatic ONFH was directly associated with steroid hormones in 47.4% of cases [4]. Steroid hormones induced ONFH has always been one of the most serious orthopedic diseases. There are many possible pathogenesis and risk factors, among which oxidative stress disorder may be one of the most common factors involved, and the oxidative stress caused by long-term use of glucocorticoids can weaken the repair capacity of bone tissue [5, 6]. Oxidative stress refers to a state where the balance between the production of prooxidants (free radicals or reactive oxygen species (ROS)) and the elimination of antioxidants is disturbed in cells; in recent years, a number of studies have shown that oxidative stress can cause apoptosis through mitochondria-dependent pathway [7]. Previous studies generally believe that ROS accumulation can induce osteonecrosis in ONFH [8]. Increasing evidence suggests a critical role for osteogenic cell apoptosis in steroid hormones induced ONFH [9]. While ROS is involved in the steroid hormones induced apoptosis of the osteogenic cells [5].

Treatment of steroid hormones induced ONFH remains an orthopedic problem, with hip replacement being the only treatment option at the end of phase 1, affecting over 10,000 new patients each year in the United States, accounting for 10% of total hip replacement (THAs) [10]. Considering the serious consequences and economic costs caused by ONFH, it is necessary to find new treatments. Recently, stem cell transplantation has been used for the treatment of early hormonal ONFH [11]. However, studies have found that the transplanted stem cells undergo a large amount of stress-induced apoptosis and senescence in the osteonecrosis area, resulting in reduced stem cell survival, limiting the effectiveness of mesenchymal stem cell (MSC) transplantation [12, 13]. Exosomes as cell-free therapy can avoid the transplantation efficiency of the cells themselves, thus gradually replacing the application of MSCss in the field of regeneration. Liu et al. [14] found that MSC-derived exosomes have repair effects on hormonogenic ONFH. However, the low yield of MSC-derived exosomes and the complex extraction methods partly limit their clinical use. Exosome mimetics vesicles (EMVs) were successfully prepared by continuously pressing cells by Jang et al. [15]. Zhang et al. [16] using the method of continuous extrusion cells successfully prepared MSC-derived EMVs (MSC-EMVs), and at the same time with MSC-derived exosomes made a comprehensive comparison, found that both in size, morphology, and composition is highly similar, but the production of EMVs is much higher than exosomes, suggesting that EMVs have great potential to replace exosomes used in regenerative medicine. MSC-EMVs were found to repair spinal cord injury as well as heart injury [17, 18]. However, whether MSC-EMVs can repair hormonal femoral head osteonecrosis remains unknown.

At present, there is still a lack of effective therapeutic strategies for ONFH, and the mechanism of glucocorticoid-induced ROS accumulation and apoptosis in osteoblasts is not fully defined. Understanding the mechanism of glucocorticoid-induced ROS accumulation and apoptosis in osteoblasts may help us to find new therapeutic directions. Therefore, this study used dexamethasone-induced osteonecrosis as an in vitro model, combined with transcriptome sequencing technology, aiming to explore the specific mechanism of ROS accumulation in dexamethasone-induced apoptosis of osteoblasts. Furthermore, we aimed to clarify whether EMV could inhibit osteogenic cell apoptosis by regulating ROS levels, in order to provide potentially viable therapeutic measures for hormone-induced ONFH.

## 2. Materials and Methods

### 2.1. Cell Culture

Human umbilical cord MSCs (HucMSCs) (cat. no. PCS-500-010) were provided by Chongqing Stem Cell Biotechnology R&D Base, Chongqing, China. HucMSCs were cultured in DMEM /F12 medium supplemented with 10% fetal bovine serum (FBS) and 1% P/S (streptomycin double antibody). Osteoblast MC3T3-E1 was purchased from the official website of the Shanghai Cell Bank, and were cultured in pecific medium (Procell, stock number CM-0378) which contains *α*-MEM (PM150421), 10% FBS (164210-50), and 1% P/S (PB180120). All cells were incubated in 37°C, 5% CO_2_ in air.

### 2.2. Preparation of hucMSC-EMVs

HucMSCs were collected, and resuspended in PBS, then extruded using a micro extruder with polycarbonate membrane flters with various pore sizes (10, 5, and 1 *µ*m) (Avanti Polar Lipids). The extruded samples were collected for ultracentrifugation at 100,000 × *g* for 1 hr at 4°C, and finally the precipitates were collected, resuspended using PBS, and filtered through 0.22 *µ*m filters. The specific step of preparing hucMSC-EMVs is the same as the anterior [16].

### 2.3. Characterization of hucMSC-EMVs

We used transmission electron microscopy to observe the morphology of hucMSC-EMVs, used nanoparticle tracking analysis to detect the size of hucMSC-EMVs, and used western blot to detect the surface markers of exosomes (CD63, Alix, and TSG101). The specific method was introduced in the previous work [16].

### 2.4. Cell Viability Was Determined by Cell Counting Kit-8 (CCK-8)

Three thousand MC3T3-E1 cells/well were plated to 96-well plates, and the cells were routine cultured for 24 hr until fully adherent, and then were treated with different concentrations of DEX added (0, 0.05, 0.1, 0.25, 0.5, 0.75, 1,2, and 4 *μ*M) for 48 hr. The medium was then discarded, 10 *μ*l CCK-8 working solution and 90 *μ*l fresh medium were added, and incubation was continued for 2 hr at 37°C, followed measuring absorbance at 450 nm using a microplate reader. After the intervention concentration of DEX was determined, MC3T3-E1 cells were also added to 96-well plates, and the cells were routine cultured for 24 hr until fully adherent, and then different concentrations of hucMSC-EMVs (0, 6.25, 12.5, 25, 25, 50, 75, 100, 200, and 400 *µ*g/ml) were added, then DEX was added 6 hr later, after 48 hr of incubation, CCK-8 working solution was added as described previously, and the absorbance was measured after 2 hr. On the issue of the timing and dose of DEX and hucMSC-EMVs, we referred to the previous studies and selected a significantly different concentration as a subsequent intervention dose based on the results of CCK-8 assay [19–21].

### 2.5. RNA Sequencing and Transcriptomic Analysis

To elucidate the specific mechanism of dexamethasone-causing apoptosis in MC3T3-E1 cells, we sent DEX-treated MC3T3-E1 cells and control MC3T3-E1 cells samples to Lianchuan Biological Co., Ltd. for RNA sequencing, and we further screened differentially expressed genes between DEX-treated and control cells, followed by KEGG enrichment analysis of differentially expressed genes. The specific steps are mentioned in the previous literature [22].

### 2.6. hucMSC-EMVs Endocytosis Experiments

hucMSC-EMVs was labeled with the membrane dye PKH26 according to the manufacturer's instructions, using 800 *μ*l EMVs (1 mg/ml), added with 1,000*μ*l DiluenC, vortexed for 1 min, added 5 *μ*l PKH26 solution, incubated at 37°C for 15 min, stopped staining, and used 100,000 *g*, 4°C for 70 min. The precipitates were resuspended with the culture medium. MC3T3-E1 cells were seeded in 24-well plates containing cell sheets and then added PKH 26-labeled EMVs at a concentration of 100 *µ*g/ml. After 0, 12, 24, and 48 hr incubation, cells were washed with PBS, fixed in 4% paraformaldehyde, stained with DAPI, and photographed under a fluorescence microscope (Nikon, K10587, Japan).

### 2.7. hucMSC-EMVs and N-Acetyl L-Cysteine (NAC) Treatment

NAC was dissolved in PBS, and 100 *µ*g/ml EMVs [21], and 5 mM NAC were selected to treat MC3T3-E1 cells based on our previous experimental results [23].

### 2.8. Flow Cyte Apoptosis

Apoptosic cell death was measured using an Apoptosis Detection Kit (BD, SanDiego, CA). In brief, DEX, EMV, and NAC were intervened in MC3TC-E1 cells for 48 hr. After the cells were digested with trypsin without EDTA and washed with PBS, they were resuspended in binding buffer and stained with annexin V fluorescein isothiocyanate (FITC) and PI. The mixture was incubated at room temperature for 15 min, and cells were analyzed by flow cytometry. Specific steps were performed as described in the by Puppel et al. [5]. Apoptotic cells were counted by flow cytometry (BD Biosciences, Franklin Lake, NJ), and the annexin V/PI ratio reflects the percentage of apoptosis.

### 2.9. Western Blot

Cellular proteins were extracted using radioimmunoprecipitated RIPA (HY-K1001, MCE) reagent supplemented with a 1% protease inhibitor cocktail (HY-K01010, MCE). Protein concentration was determined by using a BCA Assay Kit. The method of western blot is the same as our previous work [16], image acquisition and densitometry analysis were performed using ImageLab (version 6.0.0, USA). The antibody information used is placed in Table S1.

### 2.10. ROS Level Measurement

We measured the ROS levels of MC3T3-E1 cells following the protocol of the ROS Assay Kit (S0033S, Beyotime Biotechnology, China). MC3T3-E1 cells were plated in a 6-well plate at a density of 200,000 cells per well. The specific detection method of ROS is as reported by Wei et al. [24].

### 2.11. Statistical Analysis

Statistical analyses were performed using the graphics pad Prism 8.0 (GraphPad software, San Diego, CA, USA). All data are presented as the mean ± standard deviation (SD). Comparison between two groups was performed using an independent sample *t*-test and between multiple groups using one-way analysis of variance (ANOVA). *p*-Values < 0.05 represents statistically significant results. All the experiments were performed more than three times.

## 3. Results

### 3.1. DEX-Induced Apoptosis of MC3T3-E1 and Increased ROS Level

**Figure 1:**
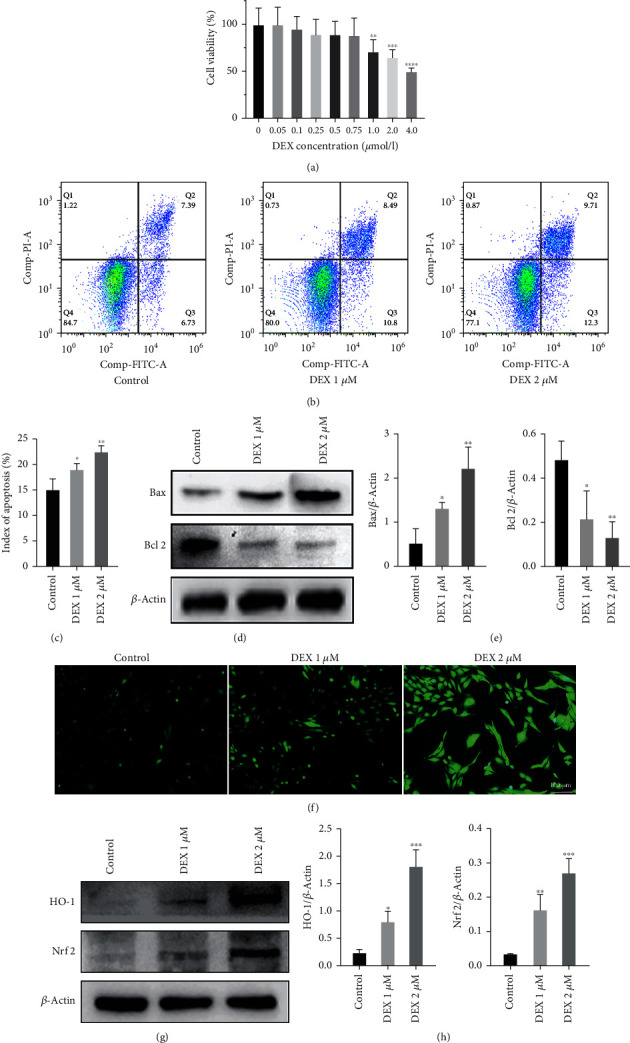
DEX induced apoptosis in MC3T3-E1 cells. (a) Cell viability of MC3T3-E1 after intervention with different concentrations of dexamethasone. (b and c) Level of apoptosis in MC3T3-E1 cells after DEX intervention by flow cytometry. (d and e) Expression of apoptosis-related proteins Bax and Bcl 2 after DEX intervention. (f) ROS generation level of MC3T3-E1 cells after DEX intervention (scale bar: 100 *μ*m). (g and h) HO-1 and Nrf 2 expression after DEX intervention by western blot.  ^*∗*^*p* < 0.05,  ^*∗*^ ^*∗*^*p* < 0.01,  ^*∗*^ ^*∗*^ ^*∗*^*p* < 0.001, and  ^*∗*^ ^*∗*^ ^*∗*^ ^*∗*^*p* < 0.0001 compared wtih control group.

We first intervened the osteoblasts by dexamethasone with different concentration gradient, looking for the dose causing the altered cell viability as the best intervention dose, and we found that the osteoblasts viability decreased significantly at DEX 1 and 2 *μ*M (Figure 1(a)). We found by flow cytometry that the level of apoptosis in osteoblasts increased significantly after DEX intervention (Figures 1(b) and 1(c)), and the western blot results showed that the expression of proapoptotic protein B-cell lymphoma 2- (Bcl 2-) associated X (Bax) increased significantly and the expression of antiapoptotic protein Bcl 2 decreased significantly after DEX intervention (Figures 1(d) and 1(e)). We further examined the ROS level in MC3T3-E1 after DEX intervention, and found that the ROS level was significantly increased after DEX intervention (Figure 1(f)), and the western blot results showed that the expression level of oxidative stress-related proteins HO-1 and nuclear factor erythroid-derived 2-like 2 (Nrf 2) was significantly increased (Figures 1(g) and 1(h)).

### 3.2. Transcriptomic Analysis of MC3T3-E1 Cells from Control and DEX-Treated Mice

**Figure 2:**
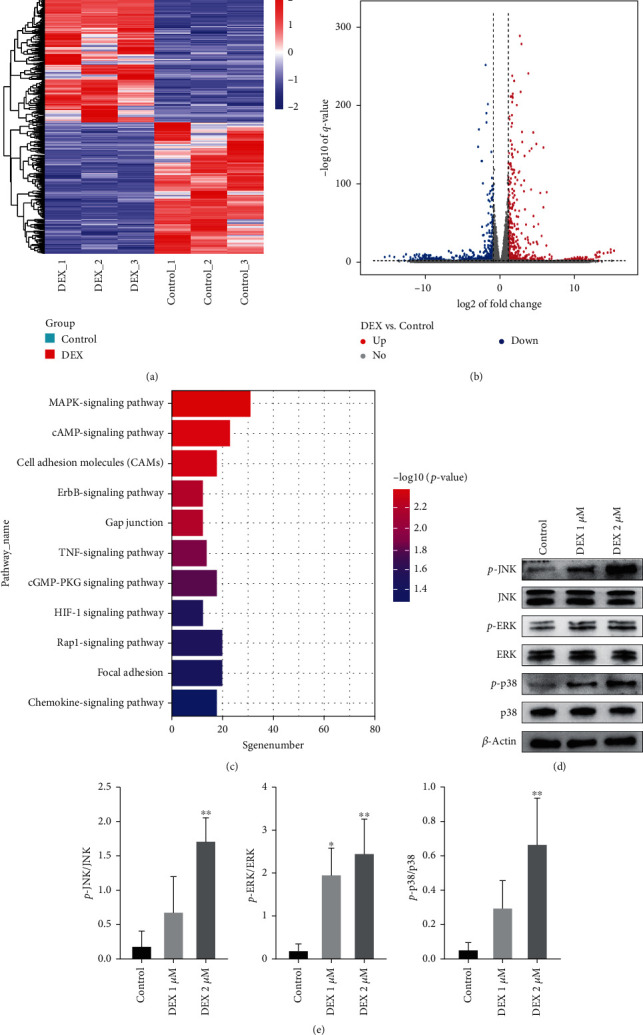
RNA sequencing analysis and pathway detection of MC3T3-E1 cells after DEX intervention. (a) Differential gene heat map of MC3T3-E1 cells between the DEX intervention and control groups. (b) Differential gene volcano plots. (c) KEGG enrichment analysis of the differential genes. (d and e) Western blot for detection of key protein expression of MAPK-signaling pathway in MC3T3-E1 cells after DEX intervention.  ^*∗*^*p* < 0.05 and  ^*∗*^ ^*∗*^*p* < 0.01compared wtih control group.

After transcriptomic analysis of MC3T3-E1 cells afer DEX 2 *μ*M intervention and control group, the heatmap of differential genes showed good clustering of the two groups (Figure 2(a)). The volcano plot showed that 1,274 differential genes were found between the two groups, of which 643 were upregulated and 631 were downregulated (Figure 2(b)). KEGG enrichment analysis of the differential genes showed significant enrichment to the MAPK-signaling pathway (Figure 2(c)).

### 3.3. DEX Leads to the Activation of the MAPK-Signaling Pathway in MC3T3-E1 Cells

According to the results of the KEGG enrichment analysis, we considered that the osteoblast apoptosis induced by DEX is likely due to the activation of the MAPK-signaling pathway. Therefore, we examined the changes of the key proteins *p*-JNK, *p*-ERK, and *p*-p38 in the MAPK-signaling pathway in osteoblasts after DEX intervention, and found that the MAPK-signaling pathway was significantly activated (Figures 2(d) and 2(e)).

### 3.4. Characterization of hucMSC-EMVs

**Figure 3:**
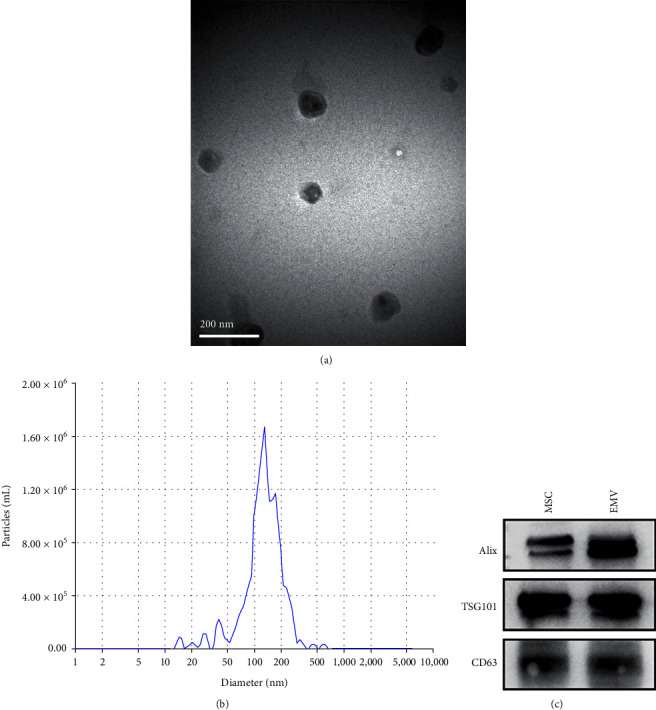
Characterization of the hucMSC-EMVs. (a) TEM showed hucMSC-EMVs as round or elliptical particles with bilayer membrane structure (scale bar: 200 nm). (b) Grain size analysis showed that hucMSC-EMVs mainly clustered between 100 and 200 nm. (c) Exsome marker protein expression by western blot.

We successfully extracted hucMSC-EMVs by continuously squeezing cells, and transmission electron microscopy showed that MSCs' vesicles were round particles with double membrane structure (Figure 3(a)), with a diameter concentrated between 100 and 200 nm (Figure 3(b)). The results of western blot showed that the exosome marker proteins ALIX, TSG101, and CD63 are equally enriched in the hucMSC-EMVs (Figure 3(c)).

### 3.5. Endocytosed of hucMSC-EMVs in MC3T3-E1 Cells

**Figure 4:**
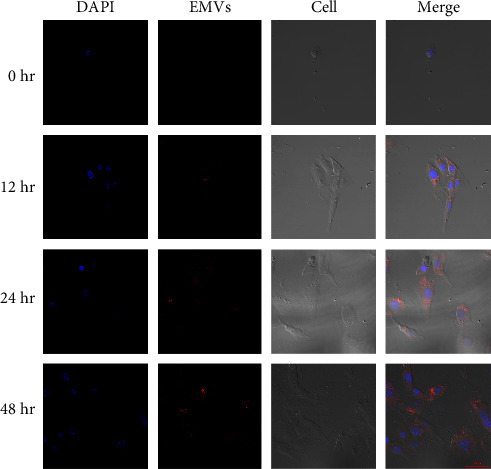
Endocytic hucMSC-EMVs in MC 3 T 3-E1 cells. EMVs were not endocytosed into the cytoplasm at 0 hr, but they were endocytosed into the cytoplasm at 12, 24, and 48 hr, and EMV endocytosis increased significantly over time, increasing aggregation along the nuclear membrane at 24 and 48 hr (scale bar: 50 *μ*m).

We examined whether MC3T3-E1 were capable of endocyting hucMSC-EMVs. The results showed that MC3T3-E1 could absorb hucMSC-EMVs; hucMSC-EMVs were not endocytosed into the cytoplasm at 0 hr, but they were endocytosed into the cytoplasm at 12, 24, and 48 hr, and EMVs endocytosis increased over time and increased along the nuclear membrane at 24 and 48 hr (Figure 4).

### 3.6. hucMSC-EMVs and NAC Attenuate Apoptosis of MC3T3-E1 Cells Caused by DEX

**Figure 5:**
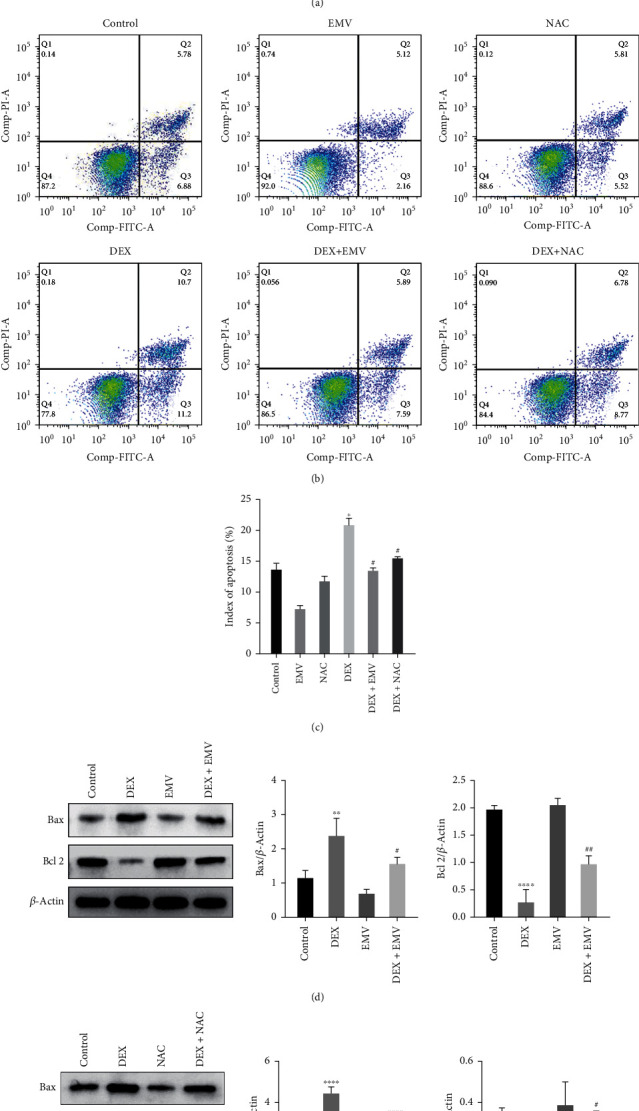
Changes in apoptosis levels in MC3T3-E1 cells after hucMSC-EMVs and NAC intervention. (a) CCK-8 test for the viability of MC3T3-E1 cells after intervention with different concentrations of hucMSC-EMVs. (b and c) Apoptosis level of MC3T3-E1 cells after hucMSC-EMVs and NAC intervention. (d) Bax and Bcl 2 expression after intervention of hucMSC-EMVs. (e) Bax and Bcl 2 expression after NAC intervention by western blot.  ^*∗*^*p* < 0.05,  ^*∗*^ ^*∗*^*p* < 0.01, and  ^*∗*^ ^*∗*^ ^*∗*^ ^*∗*^*p* < 0.0001 compared wtih control group. ^#^*p* < 0.05, ^##^*p* < 0.01, and ^####^*p* < 0.0001 compared to the DEX group.

We determined the optimal dose of hucMSC-EMVs to repair DEX-induced osteoblast injury using CCK-8, which showed that osteoblast viability increased after the EMVs dose above 25 *µ*g/ml compared with the DEX treatment group, and the difference was more significant after 100 *µ*g/ml EMVs intervention (Figure 5(a)). Therefore, the 100 *µ*g/ml EMVs intervention was selected for all the subsequent experiments. We further measured the apoptosis level of MC3T3-E1 by flow cytometry after EMVs and NAC intervention, and found that both EMVs and NAC significantly inhibited DEX-induced osteoblast apoptosis (Figures 5(b) and 5(c)). The results of western blot showed that Bax level significantly decreased after EMVs and NAC intervention compared with DEX alone group, while Bcl 2 level significantly increased (Figures 5(d) and 5(e)).

### 3.7. hucMSC-EMVs and NAC Can Inhibit DEX-Induced Elevation of ROS Levels and MAPK-Signaling Pathway Activation in MC3T3-E1 Cells

**Figure 6:**
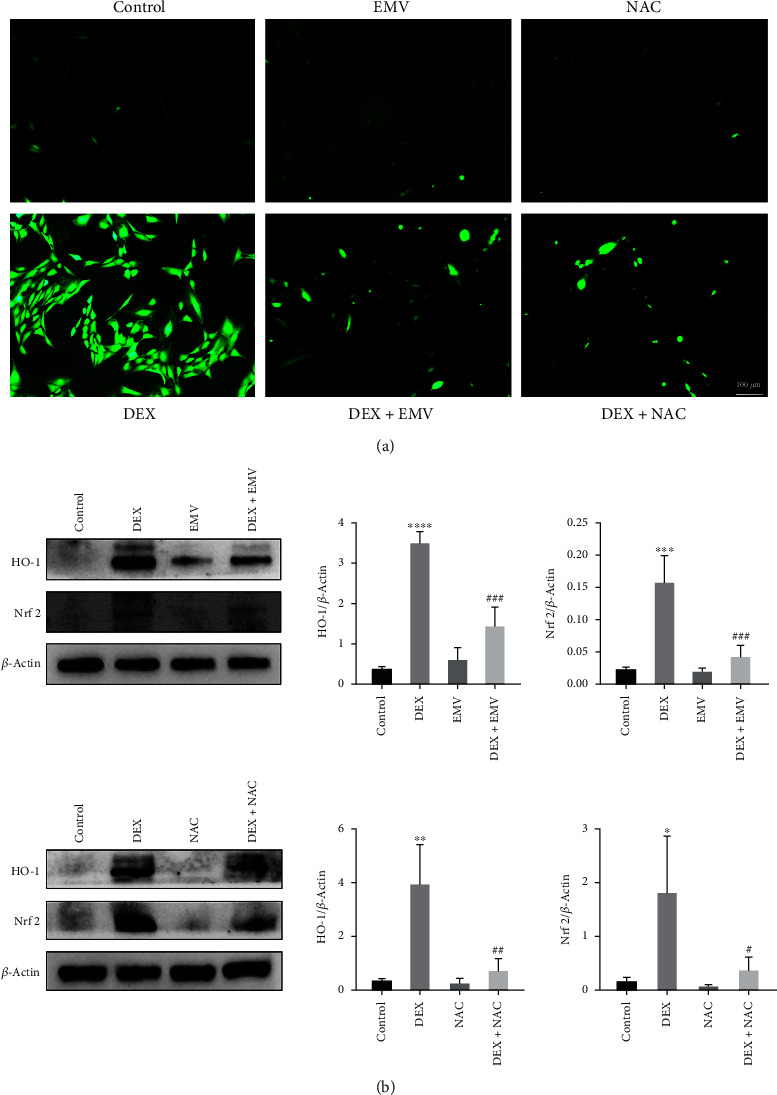
Oxidative stress levels in MC3T3-E1 cells after hucMSC-EMVs and NAC intervention. (a) ROS generation level of MC3T3-E1 cells after hucMSC-EMVs and NAC intervention (scale bar: 100 *μ*m). (b) HO-1 and Nrf 2 expression levels in MC3T3-E1 cells after hucMSC-EMVs and NAC intervention by western blot.  ^*∗*^*p* < 0.05,  ^*∗*^ ^*∗*^*p* < 0.01,  ^*∗*^ ^*∗*^ ^*∗*^*p* < 0.001, and  ^*∗*^ ^*∗*^ ^*∗*^ ^*∗*^*p* < 0.0001 compared wtih control group. ^#^*p* < 0.05 and ^##^*p* < 0.01 compared to the DEX group.

**Figure 7:**
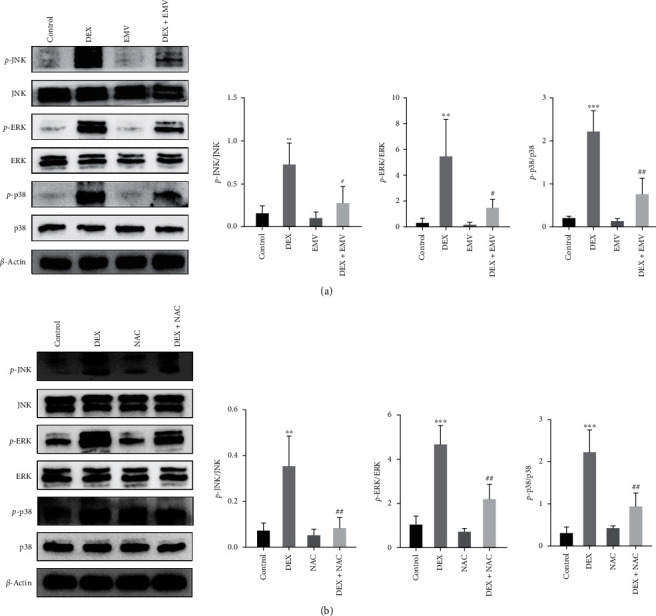
Changes in the MAPK-signaling pathway in MC3T3-E1 cells after hucMSC-EMVs and NAC intervention. (a) The expression levels of key proteins of the MAPK-signaling pathway in MC3T3-E1 cells after hucMSC-EMVs intervention were detected by western blot. (b) Expression levels of key proteins of the MAPK-signaling pathway in MC3T3-E1 cells after NAC intervention.  ^*∗*^ ^*∗*^*p* < 0.01 and  ^*∗*^ ^*∗*^ ^*∗*^*p* < 0.001 compared wtih control group. ^#^*p* < 0.05 and ^##^*p* < 0.01 compared to the DEX group.

To verify whether hucMSC-EMVs affected DEX-induced osteoblast apoptosis by inhibiting ROS level and MAPK signaling, we further examined the ROS levels after EMVs and NAC intervention, and found that EMVs could inhibit the DEX-induced ROS levels with the same effect as NAC (Figure 6(a)). The results of western blot showed that adding EMVs and NAC significantly inhibited the increase of HO-1 and Nrf 2 levels caused by DEX (Figure 6(b)). In addition, EMVs ((Figure 7(a)) and NAC (Figure 7(b)) significantly inhibited the DEX-induced activation of the MAPK-signaling pathway.

## 4. Discussion

Our study found that DEX induces apoptosis in MC3T3-E1. Further combination of transcriptomic analysis found that oxidative stress and MAPK signaling play a key role in it, and verified that DEX leads to increased ROS levels, in addition, DEX leads to activation of MAPK-signaling pathway. We also found that hucMSC-EMVs intervention significantly reduced DEX-induced osteoblast apoptosis and reducing ROS levels as well as inhibiting the MAPK-signaling pathway, with the same effect as the ROS inhibitor NAC. In brief, EMV may contribute to steroid-induced necrotic repair of the femoral head by regulating ROS levels and the MAPK-signaling pathway.

Epidemiological data suggest that the occurrence and development of ONFH are closely related to steroid hormones [25]. Long-term use or high doses of steroid hormones may cause osteonecrosis [26]. Osteoblasts play a key role in the osteogenesis process and in the prevention of osteonecrosis [27]. As a widely used glucocorticoid, DEX can promote the apoptosis of osteoblasts [28, 29]. ROS accumulation is associated with several diseases, ROS-mediated apoptosis was also demonstrated in AlCl_3_-induced dysfunction of MC3T3-E1 cells as studies by Liu et al. [30]. ROS involvement was also observed in LPS-induced apoptosis of the lung epithelial cells [31]. Several studies confirm that DEX may promote excessive generation of ROS as well as disruption of the antioxidative stress system, thereby inducing osteoblast death [32–34]. Bai et al. [6] showed that ROS was significantly increased in the DEX-induced osteoblast apoptosis, and that the ROS scavenger *N*-acetylcysteine (NAC) significantly reduced the DEX-induced ROS increase, thereby inhibiting ROS-induced osteoblast apoptosis. Our study also confirmed that DEX can promote the ROS accumulation and induce apoptosis in the osteoblasts.

DEX can induce ROS accumulation and promote osteoblast apoptosis has been confirmed in many studies, but the mechanism of DEX-induced osteoblast apoptosis is still unclear, so we further conducted transcriptome sequencing of osteoblasts and control osteoblasts after DEX intervention and screened for related pathways. We successfully enriched to the MAPK-signaling pathway by two sets of differentially expressed genes. MAP kinase (MAPK), a new serine/threonine kinase, catalyzes the phosphorylation of microtubule-associated protein 2 (MAP-2) in insulin-treated 3T3-L1 adipocytes, and extracellular signal-regulated kinase 1 (ERK 1), a cloned product of MAP kinase [35, 36]. Stress-activated JNKs and p38 MAPKs play critical roles in promoting the apoptosis [37]. Several Bcl family proteins, including the pro- and antiapoptotic groups, are already under the control of the JNK and/or p38 MAPK cascade [38]. Our study confirmed MAPK-signaling pathway activation after DEX intervention and induced changes in Bcl family proteins, with MAPK-signaling pathway activation leading to increased expression of the proapoptotic protein Bax and decreased expression of the antiapoptotic protein Bcl 2. Moreover, MAPK-signaling pathway activation is closely associated with ROS levels. Studies showed that the MAPK-signaling pathway is downstream of ROS, and ROS accumulation activates the MAPK-signaling pathway to induce apoptosis [39, 40]. NAC abolished the upregulation of *p*-ERK and *p*-JNK, key proteins in the MAPK pathway, thereby inhibiting the MAPK-signaling pathway [23]. Additional studies have shown that MAPKs can be activated by the curcumin to induce the production of endogenous ROS, thereby promoting apoptosis in cancer cells [41]. The MAPK/ERK-signaling pathway is also thought to be involved in osteoporosis [42]. Our study confirmed that DEX-induced osteoblast apoptosis may result from the increased ROS production induced via MAPK-signaling pathway activation.

To date, no single treatment has completely cured the necrosis of the femoral head. Previous studies have reported a method for autologous implanted MSCs to delay or avoid the collapse of the femoral head [43]. Coculture of MSCs with osteoblasts promotes osteoblast proliferation and osteogenic [44]. All the above studies confirmed the therapeutic effect of MSCs on necrosis of the femoral head. Moreover, MSC-derived exosomes can also reduce the risk of ONFH [14]. Liao et al. [45] found that BMSC-derived exosomes can multiply promote osteoblasts in the necrosis of the femoral head. Unlike direct stem cell transplantation, exosomes have the advantages of intrinsic homing effects, low immunogenicity, and effective molecular signaling stimulation [46]. However, exosomes, due to their low yield and complex extraction, cannot be applied on a large scale. Our previous studies have confirmed that the EMVs produced by continuous extrusion of cells has a similar structure and composition to exosomes, but its yield is tens of times theof exosomes [16]. MSC-EMVs have been used widely in the various diseases. MSC-EMVs have been used as a drug delivery vehicle for a variety of cancers, including osteosarcoma, neuroblastoma, and breast cancer [47–49]. Furthermore, MSC-EMVs showed promising results in tissue repair. For example, the combination of hucMSC-EMVs with an injectable chitosan hydrogel injected into a mouse unhealed cranial defect showed robust bone regeneration [50]. EMVs from MSCs (IONP-MSCs) combined with iron oxide nanoparticles (IONPs) reduces cardiomyocyte apoptosis and fibrosis to promote cardiac repair [18]. MSC-EMVs can facilitate repair in SCI [51]. However, the effect of MSC-EMVs on the ONFH has not been deeply studied. We demonstrated that hucMSC-EMVs reduced DEX-induced osteogenic cell apoptosis as well as ROS accumulation. Furthermore, MSC-EMVs inhibited DEX-induced activation of the MAPK-signaling pathway and had the same effect as the ROS inhibitor NAC.

## 5. Conclusions

In conclusion, our study confirmed the critical role of MAPK-signaling activation as well as ROS accumulation in DEX-induced osteoblast apoptosis. In addition, we confirmed that hucMSC-EMVs can downregulate the MAPK signaling as well as ROS levels to attenuate the DEX-induced osteoblast apoptosis. We believe that MSC-EMVs may serve as a potential treatment measure for hormonal necrosis of the femoral head. Furthermore, further in vivo studies are needed to validate the results of this study.

## Data Availability

The datasets used or analyzed during the current study are available from the corresponding author on reasonable request.

## Abbreviations

MSCs: Mesenchymal stem cell
DEX: Dexamethasone
hucMSC: Human umbilical cord MSC
EMVs: Exosome mimetic vesicles
ROS: Reactive oxygen species
NAC: *N*-acetyl-l-cysteine
NONFH: Nontraumatic osteonecrosis of the femeral head
THAs: Total hip replacement
FBS: Fetal bovine serum
CCK-8: Cell Counting Kit-8.

## Supplementary Materials

Supplementary Materials Table S1: antibodies used for different experiments in this report.
