# Supplementary material for: Human Mesenchymal Stem Cells-Derived Exosome Mimetic Vesicles Regulation of the MAPK Pathway and ROS Levels Inhibits Glucocorticoid-Induced Apoptosis in Osteoblasts
Source: Stem Cells Int. 2023 Sep 20;2023:5537610. doi: 10.1155/2023/5537610 (PMC10533242; doi:10.1155/2023/5537610)
Supplement: Supplementary Materials — Table S1: antibodies used for different experiments in this report. [file 5537610.f1.docx]

**Supplementary Table 1. Antibodies Used for Different Experiments in This Report**

| Antibody | Supplier | Catalog Number | WB |  |
| --- | --- | --- | --- | --- |
| GAPDH | ZENBIO | 200306-7E4 | 1:1000 |  |
| Bax | Proteintech | 60267-1-Ig | 1:1000 |  |
| Bcl2 | Proteintech | 26593-1-AP | 1:1000 |  |
| HO-1 | Wenleibio | WL02400 | 1:1000 |  |
| Nrf2 | ZENBIO | 380773 | 1:1000 |  |
| JNK | ZENBIO | R22866 | 1:1000 |  |
| p-JNK | ZENBIO | 381100 | 1:1000 |  |
| ERK | ZENBIO | 343830 | 1:1000 |  |
| p-ERK | ZENBIO | 301245 | 1:1000 |  |
| p38 | ZENBIO | R25239 | 1:1000 |  |
| p-p38 | ZENBIO | 310091 | 1:1000 |  |
| Alix | Abcam | 275377 | 1:1000 |  |
| TSG101 | Abcam | 125011 | 1:1000 |  |
| CD63 | Abcam | 134045 | 1:500 |  |
